# Sharing individual patient and parasite-level data through the WorldWide Antimalarial Resistance Network platform: A qualitative case study

**DOI:** 10.12688/wellcomeopenres.12259.1

**Published:** 2017-08-16

**Authors:** Elizabeth Pisani, Stella Botchway

**Affiliations:** 1 Visiting Senior Research Fellow, The Policy Institute, King's College London, London, UK; 2 Independent research analyst, Oxford, UK

**Keywords:** data sharing, bioinformatics, WorldWide Antimalarial Resistance Network, LMIC, global health, research infrastructure, incentives, research collaboration

## Abstract

Background: Increasingly, biomedical researchers are encouraged or required by research funders and journals to share their data, but there's very little guidance on how to do that equitably and usefully, especially in resource-constrained settings. We performed an in-depth case study of one data sharing pioneer: the WorldWide Antimalarial Resistance Network (WWARN).

Methods: The case study included a records review, a quantitative analysis of WAARN-related publications, in-depth interviews with 47 people familiar with WWARN, and a witness seminar involving a sub-set of 11 interviewees.

Results: WWARN originally aimed to collate clinical, in vitro, pharmacological and molecular data into linked, open-access databases intended to serve as a public resource to guide antimalarial drug treatment policies. Our study describes how WWARN navigated challenging institutional and academic incentive structures, alongside funders' reluctance to invest in capacity building in malaria-endemic countries, which impeded data sharing. The network increased data contributions by focusing on providing free, online tools to improve the quality and efficiency of data collection, and by inviting collaborative authorship on papers addressing policy-relevant questions that could only be answered through pooled analyses. By July 1, 2016, the database included standardised data from 103 molecular studies and 186 clinical trials, representing 135,000 individual patients. Developing the database took longer and cost more than anticipated, and efforts to increase equity for data contributors are on-going. However, analyses of the pooled data have generated new methods and influenced malaria treatment recommendations globally. Despite not achieving the initial goal of real-time surveillance, WWARN has developed strong data governance and curation tools, which are now being adapted relatively quickly for other diseases.

Conclusions: To be useful, data sharing requires investment in long-term infrastructure. To be feasible, it requires new incentive structures that favour the generation of reusable knowledge.

## Introduction

In recent years, some academic journals have begun to encourage or require researchers to make the data underlying published papers available to the scientific community for re-use ^1– 3^. At the same time, several major funders of biomedical research have begun to encourage or require grant-holders to share their data with other researchers ^4^.

These developments are motivated partly by a desire to maximise transparency in clinical trials and other research, and partly in the hope of speeding up discoveries that contribute to improving health. This second motivation rests on the belief that data shared will become data reused; the combination of data generated by different studies will allow for more powerful meta-analysis of complex questions; these analyses will in turn translate into better policies and practice and thus to better health, at limited additional cost. Squeezing more knowledge out of existing data is considered especially important for the often neglected diseases that affect people in poorer countries, where most research is funded by public agencies or charities, and for the infectious disease outbreaks that most commonly occur in those countries ^5^.

However, the many institutions now actively promoting data sharing give very little guidance on exactly how data should be shared in order to maximise health gains, in part because they have little systematic information on what works, what doesn't, and why. Many opinions have been aired about the potential advantages and dangers of different models ^6– 11^, and some empirical studies have examined experience with sharing data in well-resourced areas such as cancer, or cutting-edge scientific fields such as neuroimaging and genomics ^12– 17^. In most areas of tropical medicine, the potential financial rewards for generating new information through data pooling are more limited, and entrenched inequities in scientific and material resources add an additional layer of complexity. We are not aware of any published study examining in detail the experience of sharing individual patient level data generated in clinical trials conducted in low and middle income settings.

This paper seeks to begin to fill that gap by providing an in-depth case study of the development of the WorldWide Antimalarial Resistance Network (WWARN), one of the earliest networks sharing clinical trial data involving scientists from research institutions in low, middle and high income countries.

WWARN (referred to until 2010 as WARN) was an initiative of senior academic researchers working in the field of malaria who were concerned about the spread of antimalarial resistance. The network's aim was to bring together individual patient and parasite-level data in four areas - clinical, in vitro, molecular and pharmacological - to allow for the rapid tracking of and response to drug-resistant malaria ^18^. Data on the quality of antimalarial medicine was added in 2010.

The idea of bringing four different markers of antimalarial resistance data together into a single global database was first mooted in 2004 and was developed opportunistically, at informal side-meetings attached to scientific conferences which were attended by academics as well as technical experts from the World Health Organization (WHO) and other global health bodies. Clinical data were expected to include measures of drug efficacy (e.g. treatment failure by day 28 or 42) for individual patients drawn from clinical trials performed by academic researchers as well as from therapeutic efficacy studies conducted by national malaria programmes as part of their routine surveillance activities. In 2007, the Bill and Melinda Gates Foundation (BMGF) provided grants totalling close to US$ 1.1 million to the founding members to further develop the concept. BMGF invested a further US$ 20.6 million in the network in 2009, simultaneously approving a linked grant of US$7.5 million to the WHO to support surveillance of the therapeutic efficacy of antimalarials in endemic countries. That year, WWARN was established as a formal entity with a secretariat based at the University of Oxford. WWARN was conceived not as a formal consortium of research sites, but as a network of interested researchers. The scientific director was based in Seattle, while heads of the other scientific modules (clinical, pharmacology, in vitro, molecular, informatics and later medicine quality) were based, respectively, at universities or labs in Darwin, Cape Town, Paris/Phnom Penh, Oxford and Vientiane.

Using a number of qualitative methods, we examined the evolution of the network, focusing in particular on issues that facilitated or impeded the sharing of data, and the use of data that had been shared. The study, performed at the request of the Public Health Research Data Forum, was not designed as an evaluation of WWARN, nor did it intend to probe all aspects of scientific collaborations. Rather, we aimed to contribute to an evidence-based understanding of the factors that make sharing of patient-level data feasible and useful, particularly when research is conducted in low and middle income countries. A full technical report was prepared for research funders ^19^; here we focus on findings we believe to be of greatest interest to researchers who share data, or are contemplating doing so.

## Methods

The case study included a records review, a quantitative analysis of publications by WWARN and its collaborators, a series of in-depth interviews, and a witness seminar.

### Records review

For the records review, a WWARN administrator who has maintained records since the outset of the collaboration provided access to grant proposals and grant-related reporting forms; progress reports by scientific module heads, internal strategic plans and business plans; minutes of all board and Scientific Advisory Committee (SAC) meetings; iterations of terms of submission, memoranda of understanding and contracts with data providers; correspondence with the World Health Organization (WHO) and other key partners; and surveys of user and stakeholder attitudes. WWARN founders provided us with archives of conference presentations, including those made at formal and informal side meetings to malaria or infectious disease conferences. The network provided analytics data covering use of the WWARN website, downloads of tools, and social media reach. We also searched for published papers mentioning WWARN.

These documents were read by two investigators and evaluated for relevance to the experience of data sharing. Salient information was consolidated into a timeline. Documents considered to be highly relevant by either investigator were processed as below.

### Quantitative analysis of publications

All papers published using data from the WWARN repository as well as those listed on the WWARN website's "Impact" page ( http://www.wwarn.org/impact/publications) to end-June 2016 were entered into a database. For each paper we included the type of paper (coded by SB), its citation count, the regional and institutional affiliation of first and last authors, if any (some of the pooled analyses were published in the name of the study group as a whole) and the total number of authors on the paper.

### In-depth interviews

The principal investigator (EP) conducted interviews, generally of 60–90 minutes in length, with individuals purposely selected to give a wide variety of perspectives on WWARN's evolution. Potential interviewees were contacted by email with information about the purpose of the study and the broad areas of questioning to be covered, and invited to participate. Those who agreed were provided with more detailed information prior to the interview, and signed consent forms (protocol available at doi: 10.7910/DVN/V1TKIO ^20^). Interviews were conducted in English, French or Indonesian, face-to-face or via Skype, between May and July 2016.

Comprehensive notes were taken during all interviews, and where consent was given for recording, interviews were audio-recorded.

### Witness seminar

A subset of interviewees participated in a witness seminar, a format which encourages debate about how and why a recent set of events evolved as they did. This collective reflection often prompts memories and spotlights issues which don't arise in individual interviews; it provides an opportunity to validate or revisit data gathered in those interviews ^21^. The participants were purposively selected to reflect a combination of institutional memory and current experience, as well as to ensure participation from malaria endemic and non-endemic countries; our choice was also influenced by opportunism and cost-effectiveness: nine of the 11 participants were already planning to gather for a conference. All participants signed forms consenting to the recording of the seminar, and the use of the data for the purposes of this study. The four-hour seminar, which took place on June 23 2016, was chaired by two independent researchers, who were provided with a detailed issues brief by the study PI (EP). It was recorded and transcribed by a third party.

### Data handling

Notes and relevant documents from the records review, as well as the notes or transcripts of all in-depth interviews and the transcript of the witness seminar were entered into NVivo software Version 11.3.2. (QSR International) and coded thematically by the principal investigator (EP). The coding tree, which provides both our coding categories and brief descriptions of the areas they cover, can be downloaded at doi: 10.7910/DVN/V1TKIO ^20^. High order codes were deductive, derived from the original study protocol, which can also be found at doi: 10.7910/DVN/V1TKIO ^20^. These include broad topic areas such as history and evolution of the network, institutional relationships, equity and incentives. Further codes were developed inductively from the content of the data themselves (for example, under "Policies and Practice" we coded for terms of submission and access, institutional governance, informatics, data management and other relevant terms). We refined these iteratively as data analysis progressed. Thematic analysis was carried out using a modified version of the iterative categorization method described by Joanne Neale ^22^.

The study was approved by the Oxford Tropical Research Ethics Committee (OxTREC Reference: 593-16). More details related to methods are provided in COREQ file at doi: 10.7910/DVN/V1TKIO ^20^, which follows the COREQ guidelines for reporting qualitative research ^23^.

## Results

### Data and participants

We identified over 685 documents or presentations relating to WWARN, its history and evolution. After removing multiple drafts and near-duplicates and filtering for content most relevant to data sharing, we entered approximately 13% of all documents into NVivo for detailed coding.

Our database of academic publications by the WWARN network and WWARN-related papers by collaborators included a total of 77 papers; 18 of these were publications analysing data contained in the WWARN database, which we term "core" WWARN publications.

We interviewed 47 individuals. Many research participants with functional roles such as science group head, scientific advisory committee member or board member also serve on advisory boards for treatment guidelines and other policy issues at the national or international level, and most also contribute data to the database. Many other data contributors similarly have dual roles as policy advisers.

Table 1 groups respondents by their most formal relationship to WWARN, if any, otherwise by their primary professional affiliation. Thus, a WWARN board member who is also a policy maker is listed as a board member, while a researcher who contributes data to the WWARN database and also sits on a malaria control programme advisory panel is listed as a data contributor.

**Table 1.** Characteristics of people interviewed for this study.

Principal relationship to WWARN	Number of interviewees
Current WWARN staff/consultant	5
Former WWARN staff/consultant	7
Current or former scientific leaders or group head	5
WWARN board members	3
WWARN scientific advisory committee members	2
Data contributors from industry	2
Other data contributors	3
Malaria researchers who do not contribute data	3
Secondary user or analyst	4
Current or former global health organisation staff	11
National policy maker	2
**TOTAL**	**47**

Twenty-one of the interviewees were women, and 11 were from low or middle income countries. Some 46% were based in Europe at the time of their involvement with WWARN, with the remainder evenly split between Asia/Australasia, Africa and North America.

Eleven of the interviewees also participated in the witness seminar. Of these, four were women, and four were from low or middle income countries. They comprised three current WWARN staff, three board or SAC members (two of whom are active data contributors), three science group heads, one former employee of a global health organisation and one other data contributor.

No-one refused participation point-blank, and no-one who engaged in an interview terminated it. However five individuals failed to respond to repeated approaches by e-mail: two former employees of WWARN; one current and one former employee of the World Health Organization; and a policy-maker in a malaria-endemic country. A further two individuals, both senior researchers based in malaria-endemic countries, agreed to be interviewed but were unable to make any of the repeatedly-scheduled interview times. Three participants scheduled to participate in the witness seminar did not participate, one for personal reasons and two because of difficulties getting visas to the UK.

### The early phase of WWARN's development

***Early intentions.*** In 2006, Carol Sibley, a professor of genome sciences at the University of Washington, and Pascal Ringwald, a WHO official responsible for surveillance of malaria resistance, published a paper advocating for the development of a shared data resource to track malaria resistance. They envisaged:

*“[…] a dynamic, open access database that would include current and historical data on clinical efficacy, in vitro responses and molecular markers related to drug resistance in Plasmodium falciparum and Plasmodium vivax. The goal is to include historical and current data on resistance to commonly used drugs […] and on the many combinations that are now being tested in different settings. The database will be accessible to all on the Web.”* ^18^

A year later, a series of four papers published in Malaria Journal envisaged a " *comprehensive efficacy and resistance database [which] will provide malaria control managers, surveillance programs and policymakers with prompt access to up-to-date evidence of temporal and geographic trends in antimalarial drug resistance at the global scale*." ^24^

In many early presentations, promoters of the concept described the venture as a public good which would allow policy-makers to take quick action to avert or limit treatment failure by adapting antimalarial regimens as necessary.

***Meeting different needs.*** Among interviewees, well-established academics from wealthy countries and the staff of global health organisations were particularly likely to see the global tracking of resistance as an important and useful goal. Several used the term "no-brainer" in describing the project. In other words, they believed the rapid compilation of research and surveillance data would self-evidently add value to the research itself, because it would allow policy-makers to see the "big picture" sooner and more easily. Policy-makers in endemic countries were less likely to see a global database as helpful. Those we spoke to said they tended to look to the WHO for guidance on drug efficacy - a point also mentioned by many endemic country researchers. Further, programme managers said most of their concerns were localised and operational.

*“The other thing that comes up with local stakeholders is the issue of relevance. Say I'm in an area with a lot of resistance to a drug, and you analyse pooled data, lots of which were collected in an area where resistance to that drug is not an issue, what sort of information will you be providing? How will local confounders be dealt with when you have millions of data points, the majority of which are irrelevant to your own setting?”*

[I28, Endemic country policy maker]

Although researchers from the same country confirmed that policy-makers tended to give great weight to local studies, they noted that cross-country meta-analyses also had a role in shaping programme managers' opinion, in two rather less direct ways. Firstly, national researchers often drew on WWARN analyses to inform the questions they asked locally, and to contextualise and add weight to their own site-specific findings when presenting them to policy makers. Secondly, analyses of pooled individual patient data have fed directly into the WHO guidelines, which are so influential in malaria-endemic countries.

National malaria programme managers are referred to as a core constituency in all of WWARN's foundational documents, but there's little recorded evidence of direct consultation with them about their needs. In October 2006, in the first meeting called expressly with a view to discussing WWARN, presentation notes record " *Inform policy making process*" as the first aim of the network, with the note " *WHO has more command of this than researchers – [they] need to be on board if Africa is to accept.*"

In meetings with WWARN held to discuss the linked grants provided by BMGF, the WHO repeatedly stressed its role as the bridge between the network and national programmes. A memorandum of understanding between the two organisations signed on June 10 2009 and provided to BMGF effectively barred WWARN from working directly with national malaria programmes. One provision specifically denies WWARN permission " *to interfere in, or duplicate, in any way, WHO's role … with respect to: (a) monitoring drug efficacy (in vivo); (b) use of evidence for the generation of global treatment policies: and (c) technical advice to countries to update national treatment policies.”*

As a result, direct consultation with national policy makers was limited. In a meeting of the Scientific Advisory Committee (SAC) as late as June 2010, one endemic country researcher commented:

*“Our group is weighted towards scientists. But our purpose is to do something about malaria. We’re waiting until quite late to figure out how to make the information useful to decision makers*.”

(D48, Scientific Advisory Committee member)

In the same meeting, a WWARN science group head commented:

*“What we decide is important, may be completely irrelevant to our target stakeholders. We do need to determine stakeholder needs, to ensure that we don’t just build a resource for ourselves.”*

(I12, WWARN science group head)

***Foundational decisions about data submission.*** Minutes of project meetings show that in Oxford, an informatics team began to develop a database, including data intake and standardisation tools. They worked very largely with post-publication datasets provided by WWARN associates (mostly science group heads, SAC or board members) who had well established positions in globally recognised academic institutions or who came from non-governmental organisations.

In addition to gathering and entering patient-level data from published research studies, the intention was ultimately to populate the database with patient-level information from two additional sources: on-going (as yet unpublished) clinical trials or molecular studies, and treatment efficacy surveys carried out by national malaria programmes as part of their routine surveillance efforts. Some of the treatment efficacy surveys were funded by BMGF through a WHO surveillance support scheme which Foundation staff said aimed specifically to improve the quality and volume of data available for pooled analyses.

The effort to gather data from researchers in endemic countries was led by WWARN regional staff based in Bangkok, Nairobi, Dakar and Sao Paulo. They contacted authors of published studies and their colleagues, asking them to contribute research datasets, including individual patient or parasite-level data, to the network.

In countries where WHO was supporting resistance surveillance with funds from BMGF, the global body made the same request to national malaria programmes. WHO asked governments to sign a letter giving express permission to share data from therapeutic efficacy studies carried out for surveillance purposes with WWARN; the WHO also specifically renounced all responsibility for any loss or damage caused by Oxford's handling of national data or specimens.

The result of all of these efforts was disappointing, according to many interviewees, with very few national programmes or individual researchers choosing to contribute information to the resource:

*“We were not exactly flooded with data*.”

(I12, WWARN science group head)

***Historical context: data sharing professionally unrewarding.*** All interviewees underlined the historical context. By the time WWARN was set up, funders had begun to mandate sharing of data in the field of genomics, and demographic and health data collected under contract for programmes such as the Demographic and Health Survey were also routinely anonymised and shared. However, there were still very few examples of successful sharing of individual patient data in clinical medicine, especially in lower-income settings, and virtually none outside of closed consortia. According to one interviewee, many researchers were viscerally opposed to data sharing:

*“Seven years ago, "data sharing" was a swear word*.”

(I31, Current WWARN employee)

All WWARN's early documentation described the shared resource as "open access". At the time, the phrase was used largely to refer to making scientific papers freely available to anyone with an internet connection. The 2009 grant application stated that research tools, as well as the output of any mapping, analysis or other research using the database, would be openly available to all-comers. Although WWARN founders said in interviews that the intention was never to share individual level data openly, this may not have been obvious to the endemic country investigators who had data to contribute. Draft terms of submission began to circulate in 2010, though they were not finalised and formally available to potential contributors until March 2011. This was in part because of the considerable time that it took WWARN secretariat staff to persuade Oxford University lawyers that seven pages of often arcane legal language could be streamlined into a three-page document in plain English, understandable to malaria researchers worldwide.

Once the terms of submission were published, it became clear that access to data submitted to the WWARN database was restricted to the group submitting the particular dataset and to WWARN employees, who would only share the aggregated results of any analyses they performed. The earliest Terms of Submission added: "In the event that WWARN receives a request to contribute data to an external project or collaborative analysis, it will not contribute or grant access to your data without your express permission."

Despite these restrictions, many interviewees, including several who did eventually contribute data to WWARN, were initially reluctant to provide research results to the database. By far the most common reason given, especially among respondents from malaria-endemic countries, was the fear that WWARN researchers based at Oxford or other well-resourced universities would analyse their data and publish results before they themselves had time to get out of the clinic and write up their findings. Though all interviewees (including those who chose not to share data with WWARN) recognised the contribution that data sharing might make to global surveillance of malaria resistance, most were not initially prepared to risk being "scooped", thus sacrificing personal, first-author publications and the chance for academic advancement.

*“If you've shared the data there's a feeling that, well, you never get it back… You feel like you have lost something. It's a global good, everyone is seeing that, but you've lost it as the person who got the study going.”*

(I19, Endemic country researcher, data contributor)

Others chose not to contribute data at all, because WWARN did not pay for data collection. The network did develop and share data collection and analysis tools, and provided some funding for training in analysis and other capacity building activities, but some felt the “carrot” was simply not big enough.

*“Where do they expect me to get the resources to go and conduct prospective studies? You can't expect me to hand my data to you if you didn't even support me to collect it […]. If I know you have 21 million dollars and you say all you can do for me is invite me to put it in some database and then show me how to do some meta-analysis, well, I'm not impressed*.

(I20, Endemic country researcher - not data contributor)

Researchers were also concerned that the curation process might reveal weaknesses in the data, potentially calling into question published analyses. Interviewees in industry were most worried that re-analysis might yield results that differed slightly from those used in product registration, while some policy-makers had concerns over data ownership.

*“For the national programmes, the question of data ownership is very real. More than once I've heard people say: why are we giving African data to England? So you still need a lot of discussions with countries, so that they don't think their data are being “stolen” by the north*.”

(I9, Former WWARN employee)

The WHO, the most important bridge between national programmes and the wider research community, could perhaps have helped in those discussions. The global body wrote several letters supporting the idea of the network, and several WHO experts, including those responsible for malaria resistance surveillance, contributed to the planning and development of WWARN. The arrangement highlighted the extent to which WHO relied on “soft” money, such as grants from BMGF and other donors, to carry out its normative functions.

*“In the malaria department like all the disease specific ones, the Member States provide less than 20 % of the budget. [The department] has to go out getting money, so [is] not so different from academic institutions. But [the WHO has] all the limitations of having to answer to all of [the] Member States, to follow all the protocols and regulations, to conform with other UN institutions.”*

(I07, Global health organisation employee)

The 2009 memorandum of understanding between WHO and WWARN was not enough to reassure technical experts within WHO. They had been working for many years to support surveillance of malaria treatment failure on a shoestring budget, and they felt undermined by the surge of resources available to WWARN.

*“When [WWARN] started to get lots of money[…] they started to think they could duplicate WHO work, normative and surveillance, and progressively replace this activity in WHO, take it over and run it on their own.”*

(I01, Global health organisation employee)

Virtually all interviewees said that the relationship between the two organisations was scarred as a result of these rivalries. The tension passed down to WHO country offices, which work closely with national malaria control programmes. According to a senior malaria adviser to several health ministries:

*“It is true that WHO also influenced NMCPs [national malaria control programmes] not to share the data.”*

(I17, Global health organisation employee)

***Technical issues in data sharing.*** Important technical decisions had to be made in the design and construction of the database. Many of these significantly affected the pace at which the collaboration proceeded.

Interviewees described the following debates:

**1. Pre-definition of the purpose and end-use of the database**

Data scientists and informatics staff favoured a clear description of the end-use of the database at the design stage; malaria scientists, wishing to maximise the possible uses of this as yet untested resource, preferred to avoid any definition that would foreclose possible future uses. They advocated maximum flexibility, and resisted a tight, purpose-driven design.

**2. Heterogeneity of data types**

By design, WWARN intended to include clinical, in vitro, molecular, pharmacological and (later) drug quality data. The most heterogeneous of these were the clinical data. The variables defined by the WHO's resistance monitoring protocols, which were already standardised, formed the core. However there was considerable debate about which other data should be included. For other variables, such as in vitro, different research groups were measuring similar indicators using different methods or definitions. At the time, no agreed standards existed and they needed to be developed (something that has since been achieved, as noted below).

**3. Heterogeneity of data formats**

WWARN aimed to bring together information from many individual research groups, each of which stored their data in different file formats or using different metadata, even for indicators measured in the same way. Some data scientists, notably those with experience building genomics databases in which free sequencing had been offered to data contributors, advocated requiring data contributors to standardise data formats before submission. Others felt that this would discourage the contribution of clinical data (which tends to be more complex than genomic data), especially in a setting where database managers could not offer free sequencing or other similarly attractive incentives in return.

**4. Inclusion of retrospective data**

Information scientists noted that retrospective data tend to be more varied in content and format than data gathered with sharing in mind, while also being ill-suited to meeting the original goal of the database, which was to allow for real-time surveillance of resistance. Malaria scientists, on the other hand, thought excluding retrospective data would restrict the value of the resource. In addition, few researchers were prepared to contribute prospective, prepublication data; without retrospective data it would be hard to arrive at a proof-of-concept.

**5. Terms of submission**

Terms of submission and use seek to clarify the rights and responsibilities of data contributors and of secondary analysts. More restrictive terms of use, for example the obligation to seek the express permission of the contributor for every re-use of the data, tend to reassure contributors, while increasing the workload of data managers. While some interviewees (principally in global health organisations) were philosophically in favour of open access to data, most acknowledged that more restrictive conditions might be necessary to promote sharing in an environment in which sharing was virtually unknown.

**6. Curation on submission or on demand**

The more heterogeneous the data, the greater the burden of curation - of checking data validity, completeness and consistency. This raised the question of whether all variables in a dataset should be curated at the time of contribution, or whether data should be ingested but some of the curation work should be postponed until demand arose for a particular variable to be used in analysis.

Table 2 summarises the choices that data scientists were faced with when setting up the WWARN database; the grey rows indicate the choices that the network ultimately made. As the table shows, WWARN in most cases chose options that would maximise the likelihood that researchers would contribute data, as well as allowing for the greatest possible range of uses of data in secondary analysis. The options that delivered the most complete, useful database were, however, never the fastest or the cheapest.

**Table 2.** Design issues considered when setting up individual patient databases. PD: Purpose-dependent. Fields shaded in grey indicate WWARN’s choice.

Issue	Choice	Minimises		Maximises	
		Cost	Time	Data contribution	Research Utility
**End Use**	Clearly pre-defined	✓	✓		
	Flexible design				✓
**Heterogeneity of** **data type**	Homogenous	✓	✓		
	Heterogeneous			✓	✓
**Heterogeneity of** **data format**	Standardised before intake	✓	✓		
	Standardised after intake			✓	
**Age of data**	Prospective only	✓	✓		PD
	Retrospective data also included			✓	PD
**Terms of** **submission**	More open	✓	✓		✓
	More restrictive			✓	
**Time of curation**	On demand *	✓	✓		
	At intake				**?**

* WWARN's practices on curation vary - core variables are curated at intake, others, often those used for Study Group analyses described below, on demand.

This meant that progress in constructing the database was inevitably rather slow, which came as little surprise to interviewees who had been involved in similar projects in different fields:

*“At [an HIV data sharing consortium] it took us a good seven years to build relationships, to get people to want to contribute their data, and this was, like, 18 guys who already knew each other.”*

(I03, Former WWARN employee)

However, the time invested in the relationships and structures needed to build a secure and useful data platform led to some frustration on the part of global health bodies who had hoped that data sharing would yield quick insights into antimalarial resistance.

*“WWARN was running for three years and there was no product; from a funder's point of view it's understandable that people say ‘OK, you're not delivering’".*

(136, Current WWARN employee)

These choices, and the standards-setting and tools development that flowed from them, were especially time-consuming for the network's largely unpaid science groups heads and other senior malaria specialists.

*“There are people who put in months of time, including my time, it was a massive sink of time.”*

(I12, WWARN science group head)

### WWARN refocuses the scientific collaboration

Table 3 provides a timeline, derived from the records review, of milestones in WWARN's development. Interviewees were unanimous in saying that by mid-2010, it had become apparent that WWARN was unlikely to develop successfully into a real-time surveillance tool that would be used by national programmes. The database was functional, and included 25,000 patient records from 78 studies, 64 of them provided by just three research groups who were enthusiastic supporters of the WWARN project. The longer-than-expected design phase had combined with professional disincentives and institutional and personal rivalries to create barriers to expansion that were simply too high to overcome.

**Table 3.** WWARN Timeline, derived from records review. DP: dihydroartemisinin-piperaquine; AS-AQ: artesunate –amodiaquine; AL: artemether-lumefantrine; WHO/TDR: The World Health Organization/Special Programme for Research and Training in Tropical Diseases.

Date	Events
**2004 – 2007**	Carol Sibley first presents the idea of WWARN at the 1 ^st^ Molecular Approaches to Malaria meeting, 2004. Side-meetings continued at many malaria/tropical medicine conferences.
**September 2007**	Seattle Biomedical Research Institute awarded US$1,021,401 by the Bill and Melinda Gates Foundation to begin planning WWARN.
**November 2007**	WWARN concept launched at the annual meeting of the American Society of Tropical Medicine and Hygiene.
**2008**	Oxford University chosen as a location. Philippe Guerin chosen as Executive Director.
**January 2009**	Oxford University awarded US$20,674,222 grant from Bill and Melinda Gates Foundation to implement WWARN.
**July 2009**	Memorandum of Understanding with WHO.
**July 2009**	WHO awarded US$7,828,470 grant from the Bill and Melinda Gates Foundation to support for therapeutic efficacy surveillance studies.
**2009**	United States Agency for International Development grant received by Molecular Group in collaboration with University of Maryland for training of researchers in Southeast Asia. Data Sharing Agreement drafted.
**2010**	Development of data sharing software. Consensus meeting held with WHO.
**March 2011**	Terms of Submission document finalised.
**July 2011**	Memorandum of Understanding with GlaxoSmithKline.
**October 2011**	First study groups launched: DP Dose Impact AS-AQ/AL Molecular Marker AS-AQ Dose Impact
**November 2013**	First study group paper published.
**October 2013**	Grant from ExxonMobil.
**2014**	230 partners working with WWARN. 100,000 individual patient data from 50 endemic countries. Novartis requested use of the Parasite Clearance Estimator model developed by WWARN to register new drugs. Agreement to standardise data collection on core variables, along with flexibility to collect additional variables.
**2015**	Change in board structure and membership. Board approval for development of other disease platforms.
**2016**	Provisional agreement with WHO/TDR to develop independent data access committee. Launch of the Infectious Diseases Data Observatory initiative.

In the meantime, however, the network had made solid (if not yet highly visible) progress developing key data management tools, governance structures and relationships with researchers in endemic settings. Rather than waste this important work, the network changed tack, focusing on academic research rather than real-time tracking of resistance.

***The study group model.*** WWARN science group heads and staff members began to formulate specific scientific questions that could not be answered by a single clinical trial, forming multi-site study groups. The first study group conducted a pooled analysis of lumefantrine pharmacokinetics in patients with malaria. The senior authors of 31 publications were asked to contribute specific variables to the WWARN database to answer a specific question. They were invited to collaborate in the analysis, and assured of authorship on any resulting publication. Twenty-six research groups shared their data with WWARN for the study.

*“Suddenly, eighty percent of people wanted to be part of something they couldn't do on their own, because they saw the real value there.”*

(I04, WWARN science group head)

The early 'study group phase' acted as a proof-of-concept for data contributors and for users alike. As more pooled analyses were published, apparently without the damage to careers that endemic country researchers first feared, data managers said they saw contributions to the database increase.

*“People now say yes to study groups much more quickly than they did in the beginning. The DP* [ *Dihydroartemisinin-Piperaquine*] *study group was two and a half years from first call going out to publication. The gametocyte study group was 12 months.”*

(I45, Current WWARN employee)

By mid-2016, the database had grown more than five-fold in terms of patient records. It now included 186 clinical trials comprising over 135,000 individual patient records. This included a remarkable 80% of all published clinical trials reporting on the efficacy of artemisinin combination therapies. In addition, the database contained 103 molecular studies. Data are contributed by academic groups, pharmaceutical companies and non-profit drug development groups such as Drugs for Neglected Diseases Initiative, as well as non-governmental organisations such as Médecins Sans Frontières.

By July 2016, 18 "core" papers using data from the WWARN database had been published, mostly developing new methods or addressing questions that could only be answered by combining data from large numbers of studies. At least two of these papers contributed directly to a change in WHO treatment recommendations ^25^. The WWARN website lists another 59 papers by researchers who have collaborated with the network. Table 4 provides information about the content of these papers; all references can be downloaded using the bibliography file at doi: 10.7910/DVN/V1TKIO ^20^). Papers considered core WWARN analyses are tagged “wwarn_core”.

**Table 4.** Content Analysis of WWARN papers. RCT: randomised controlled trial.

Type of paper	Core WWARN	Other	Total
Pooled analysis	8	0	8
Traditional meta-analysis	0	5	5
Systematic Review	1	3	4
Opinion piece or discussion article	2	18	20
RCT report or protocol	0	3	3
Other epidemiological study	1	10	11
Molecular, *in-vitro* or pharmacokinetic study	3	7	10
Modelling study	1	3	4
Methods study	2	3	5
Evaluation of equipment	0	1	1
Medicine quality	0	3	3
Other	0	3	3
	18	59	77

***Creating equity.*** Clinical trials related to malaria usually occur in malaria-endemic countries, and most are led by researchers from those countries, sometimes in partnership with research institutes in non-endemic countries. WWARN's early scientific directors were based at well-resourced universities in Seattle, Baltimore, Paris, Darwin, Oxford and Cape Town. The WWARN database is hosted by Oxford University in a secure cloud-based environment. Two interviewees external to WWARN mentioned that users physically located outside of Oxford who hoped to analyse pooled data remotely faced access restrictions. From the start, the network recognised that establishing equity would be a challenge, but should be prioritised.

*“You can't simply ignore the disparity between the resources in the north and south. If you try and bury that in the sand at the start, it will stop the project from working.”*

(I09, Former WWARN employee)

WWARN was not intended as a research funder, and did not provide funds to carry out field research. Rather, the network supported researchers from malaria-endemic countries by developing standardised clinical protocols and microscopy and other procedural guidelines. These were followed with analytic tools such as the parasite clearance estimator. Interviewees said the tools were intended to reduce duplication of effort and increase efficiency. The tools also helped researchers to collect data in standard formats that would contribute to quality, and that could be easily ingested into the database and analysed.

*“We realised in [2010] that if you didn’t provide measurement tools then people would measure things in different ways. So we started the idea of the parasite clearance estimator, because it was necessary to have a standardised way of measuring artemisinin resistance. We realised there was a bit of anarchy in the in vitro testing, so let's get a standardised measure for that... It was all very well having data, but what if they are wrong? Capacity building is not touchy-feely, it's necessary.”*

(I34, WWARN board member)

The 2009 grant application clearly described capacity building efforts that would help to support endemic-country researchers in using these tools and in analysing their data, using a regional centres of excellence model. Although the Foundation awarded the grant, its staff said they had misgivings about the approach early on.

*“I remember… being very concerned that this was going to be an increasingly expensive undertaking if we had regional, sub-regional centres. Because there seemed to be rapid growth in WWARN, in terms of its footprint, the number of people it had.”*

(W21, Former BMGF employee)

Several interviewees, including current and former WWARN staff, science group heads and current or former employees of global health organisations, including BMGF staff, said that the Foundation was strongly focused on efficient delivery of outputs, and never fully supported capacity building efforts.

*“We had concerns about WWARN trying to develop a fairly large footprint. It wasn't clear how that was going to benefit what we were trying to do… The Gates Foundation has been at best ambivalent about supporting capacity to undertake the studies in endemic countries. It hasn't been part of the Foundation's objective. … When someone in the Foundation makes an investment, they are expected to deliver. When the deliverable is data and information generated in a defined timeframe, you make your bet where you're likely to get the most yield. And that's very likely to be a known investigator and a known institution.”*

(I27, Global health organisation employee)

Staff who worked for the Foundation also said during the witness seminar that at the time that the original WWARN grant was made, BMGF procedures made it very difficult to channel money directly to endemic-country institutions which were collecting data. Project completion reports indicate that funding for most regional offices and other capacity building activities was terminated in 2013, at the insistence of BMGF.

While channelling funds to institutions in non-endemic countries heightened existing inequities in the eyes of some endemic country researchers (see quote from interviewee I20, above), others saw an opportunity in the desire of scientists from non-endemic countries to access the data they collected in their clinics.

*“What we have seen is that when we have data, some of the data we don't even think have value, [it] may have very great value to other institutions. Then you can negotiate and can get a good place on the author listing.”*

(I13, Endemic country researcher - data contributor)

However this researcher and several others expressed concerns that without more exposure to global datasets and training in complex meta-analysis, scientists from non-endemic countries would be unable to join the "big data" era. They would be increasingly consigned to the data collection end of the research spectrum, their involvement in analytic collaborations such as study groups merely tokenistic. Researchers from wealthy countries, for their part, said that strenuous efforts to involve endemic country colleagues in analysis were often frustrated because those researchers who had the capacity to contribute rarely had the time to do so.

*“They come back from Europe with their PhD, and they get made head of some institute for medical research, and that essentially ends their research careers.”*

(125, WWARN science group head)

Table 5 shows the distribution of authorship across WWARN-related papers published by July 2016. The 18 "core" papers are those based on analysis of data drawn from the WWARN database; the remainder are the other papers listed on the "Impact" page of WWARN's website. These data reflect the fact that the majority of study groups have been initiated by science group heads or WWARN, who do the bulk of the analytic work.

**Table 5.** National origin of authors on WWARN-associated publications.

	Core WWARN papers (N=18)	Other papers (N=59)
WWARN study group	7	0
Endemic country 1st author	3	13
Non-endemic country 1st author	8	46 *
Endemic country last author	0 **	4
Non-endemic country last author	11	50
Both 1st & last authors from endemic country	0	2
Both 1st & last authors from non-endemic country	11	52

*Includes 5 single author papers **One study group paper additionally includes 9 named authors. The named authors are listed alphabetically and we have thus excluded the final (endemic country) author from this count.

The seven papers 'authored' by study groups list a mean of 116 contributors alphabetically at the end of the paper (range 44–196). The list includes those who contributed data; also undifferentiated in the list are those who conceived the hypothesis, ran the analysis and wrote the paper.

Several interviewees, covering all groups and including WWARN board members and staff, were concerned that the network, whose scientific group heads are spread across four continents, would be perceived as an "Oxford data grab" because of the affiliation of the secretariat. We therefore also looked at the institutional affiliations of first and last authors. Of the 11 "core" papers not authored by groups, 10 listed first authors with Oxford University affiliations (often in conjunction with those of other institutions), and 9 of the last authors were also from Oxford. Of the other 59 papers, 29 had an Oxford-affiliated first author and 28 an Oxford-affiliated last author. For 17 of the 59 papers, neither first nor last author was affiliated with the university where the database is housed.

A spreadsheet listing all the papers and giving details of the number of authors per paper, citation counts and affiliations of first and last authors can be downloaded at doi: 10.7910/DVN/V1TKIO ^20^.

***Involvement in formulating research questions.*** In recent years, a small number of researchers in some universities in non-endemic countries have requested data for pooled analyses from WWARN, sometimes to answer questions of immediate interest to industry or global health organisations. However study group questions are rarely initiated by researchers from malaria-endemic countries other than South Africa.

Many interviewees expressed a generalised concern that the quality as well as the relevance of analysis could suffer because platforms such as WWARN enabled analysts who had no involvement in data collection to ask potentially irrelevant questions and to interrogate data without fully understanding its provenance. They underlined that this is an issue not limited to WWARN or the field of malaria, but rather, reflective of a broader structural weakness in global health research.

" *I have the feeling lately that there is a growing gap between all the brains and data crunchers in the high income countries, then the data collection is in the field.... All the biases are not even imagined, so data are being crunched a bit blindly, giving results that aren't really reliable in the end. We need more of a link between those two worlds*"

(I35, Former WWARN employee)

The feeling of being excluded by an overly global research agenda expressed by the endemic country policy-maker quoted earlier (under "Meeting different needs", I28) was shared by some endemic country researchers.

*“You need to empower people to ask questions not just relevant to WWARN, but questions that are relevant to themselves to start with and to their immediate environment. WWARN is looking from outside; some people who are in the field every day are seeing issues, and they want to answer those questions. If you feel that they don't care about your questions, well, you don't feel encouraged*.”

(I20, Endemic country researcher - not data contributor)

On the other hand, some policy-makers simply have limited experience of or interest in the science of the disease they are charged with overseeing, according to one interviewee who has worked closely with several national malaria programmes.

*“Someone comes in [as malaria programme head] for a couple of years - without even having to know a malaria life cycle - in order to step up to a higher position… At the same time for malaria programme staff, usually there is no incentive for research involvement. Some have an interest in operational research, but often, they just want to know what the findings and interpretations are.”*

(I17, Global health organisation employee)

### The future: adapting, accelerating, reproducing

The early efforts put into tools creation and data curation increased the volume of data available and the speed at which newly contributed data can be added to the database. In mid-2016, the WWARN board began to discuss changes to the terms of submission that would allow researchers to grant access to their data in perpetuity if they chose, rather than be recontacted for every use. Access requests would be considered by an independent Data Access Committee. This committee was constituted under the auspices of the WHO's Special Programme for Research and Training in Tropical Diseases (TDR) in April 2017, after data collection for this study ended ^26^.

Some feel this does not go far enough towards making data reuse a default option.

*“We have to change the conversation to: data sharing is a given. If you don't want to share you have to take action yourself to opt out, instead of putting the onus onto people to make sharing happen. If we did it the other way around, people would quickly find data sharing is not as bad as they thought and we'd make progress much faster.”*

(I43, Global health organisation employee)

Secondary analysts who have led study groups say it has saved them months or years of work seeking permissions and standardising data.

*“People have done so much work to collect the data, then WWARN works hard to standardise it; we just sweep in at the end and get this lovely clean data to work with, it's very easy for us.”*

(I14, Secondary analyst)

However their experience highlights the importance of data discoverability: an informatics issue that was not considered by WWARN's designers in the initial phase, when it was assumed that access for external users would be limited to the summary data shown on the WWARN Explorer. Discoverability - the ability of potential outside users to find the data set and easily understand what it contains - is critical if the data are to be reused by any investigator with a legitimate research question that may be addressed by data held in the resource. WWARN took care to develop data management tools that included a full audit trail for the variables that they standardised. However, it was not feasible to fully curate all of the variables that were not related to antimalarial efficacy, thus limiting the ability of potential users of the resource to discover and request access to variables of interest. A comprehensive data inventory and a data dictionary, including of as yet unused variables, is currently being developed.

***Adapting the WWARN model for other diseases in a new era.*** Many interviewees in global health organisations and industry, including some initially sceptical about WWARN, feel that the standards to which WWARN has contributed will benefit the research community more broadly. They noted, for example, the work done by WWARN collaborators to develop malaria standards for use by the Clinical Data Interchange Standards Consortium (CDISC). From 2017, CDISC metadata standards must be used for all data submitted to United States Food and Drug Administration by organisations seeking to register drugs and medical products; other regulators are expected to follow suit. As more users converge around the standards, the interoperability of data will increase and curation costs will fall.

*“The tools linked to the CDISC standards, that's all done, so let's not re-do it. That really would be stupid.”*

(I16, Global health organisation employee)

The interviewee went on to note that data governance structures developed by the network have also proven robust to the needs of pooled analyses.

*“The legal framework is an essential piece that we can't underestimate. The right to use the data, how the data are curated, how can they be published, all these aspects, it’s a very sensitive question, and I trust that WWARN has already gone through a lot of problem solving.”*

(I16, Global health organisation employee)

Several interviewees warned that the WWARN model, and the institutional structures in which the network is embedded in a university, may not be suitable for all diseases, or all uses. The study group model depends on an academic publication incentive which has little value for researchers working in government or medical charities, and which is inimical to the needs of surveillance. However, most interviewees were confident that the structures and tools developed by WWARN for malaria could and should be used as building blocks for shared clinical data platforms for other diseases of poverty.

Since the case study was undertaken, WWARN has formally launched an umbrella organisation, the Infectious Diseases Data Observatory, to facilitate this process. At the invitation of research communities working on clinical efficacy studies in other neglected tropical diseases, the team has already begun to adapt the data infrastructure, informatics tools, policies and procedures for other diseases; these efforts demonstrate the time and cost savings achieved by building on the WWARN experience.

*“Some of the tools we've developed are pretty powerful now, and we're also proving that they can be adapted relatively quickly, compared with the original development of them, to other diseases. So for instance last summer [2015] we did a pilot to develop a schistosomiasis data sharing platform, and I'd say that was pretty well advanced in just three or four months, from scratch.”*

(I45, WWARN employee)

WWARN managers said the development of functional pilot platforms for schistosomiasis, Ebola and visceral leishmaniasis has cost a great deal less than the original malaria platform. Costs vary with the volume of data and the complexity of the disease – Ebola, for example, requires data from anonymised patients to be linked over time; this had led to considerable modifications in both data structure and governance mechanisms. In general, however, set-up costs range from US$ 100,000 to US$ 900,000. These costs cover development of the data infrastructure and governance mechanisms, but do not include investment in extensive consultation among potential contributors or users, or support for research or surveillance communities to generate or analyse data, nor the on-going costs of platform management. An very important cost that tends to be neglected when budgeting for data platform development is the prodigious amount of time and expertise contributed by researchers themselves. In the case of WWARN, much of this was a pro-bono contribution made by dedicated malaria specialists with established (and securely-funded) careers.

*“We need to distinguish how [the WWARN model] works in relation to the contributors, but also in relation to very different disease questions. Infectious disease epidemiology is very different from the efficacy of chemotherapy... On top of all the money that has been spent [on WWARN], there is a lot of high quality input in time, completely unpaid... For other diseases we don't have that.”*

(I22, WWARN board member)

Many respondents noted that changes in community norms regarding data sharing relating to diseases of poverty, in part a response to pressure from funders, would reduce the high costs of consensus building that WWARN faced in its early years.

*“The fact that [WWARN] has standardised data so that everyone can learn and analyse with the same tools: that is going to be the future, however much people are conservative and reject it and are afraid of it. This will happen, so let's make it useful.”*

(I16, Global health organisation employee)

## Discussion

Our case study aimed to capture learning about data sharing from the experience of WWARN, a pioneering data sharing initiative, launched at a time when hostility to the practice was entrenched. Box 1 summarises the characteristics of a data sharing platform that has the potential to increase policy-relevant knowledge.

**Box 1: Key lessons from the WWARN experience**

**For a data sharing platform to contribute to longer, healthier lives:**

**People who collect data must be incentivised to share it**

The "greater good" argument may work with some people, such as those who work for NGOs and some governments. Where sharing data conflicts with personal or institutional incentives (for example by potentially undermining product registration by pharmaceutical companies or career progression for academics, or by exposing data quality issues) it will be resisted. Promoters of data sharing should work to change institutional incentives in ways that support sharing.

**Structural inequities in science must be reduced**

Useful data sharing may depend on local knowledge, and local knowledge must thus be actively incorporated into shared datasets, question setting and analysis. In many parts of the world with high burdens of disease, that local knowledge is produced by research systems that suffer from decades of under-investment. Unless the resulting imbalance in scientific capital is actively addressed, data sharing initiatives are likely to produce analyses of limited relevance for policy-makers in many areas.

**There must be a clear demand for the data**

Each potential user community must articulate its own needs. Academic researchers and research funders should avoid making assumptions about what policy makers want and need. A successful platform meets expressed needs.

**Relationships with key "gatekeepers" must be productive**

The global health landscape is institutionally complex: progress often depends on cooperative action by groups with different and sometimes competing interests. Particular actors (often funders or global bureaucracies) can determine whether research gets done, or its results get used, thus affecting the potential scope and likely utility of a data sharing platform.

**Investment in data curation and governance is essential and often substantial**

Data standardisation is essential for pooled analyses; key informatics decisions will determine how long it takes and how much it costs, but the costs will always been front-loaded. Standardisation tools as well as governance mechanisms can be amortised across time and platforms as tools and systems are adapted or replicated. Investors must plan for high start-up costs and, because secure and persistent data storage is also needed, for long term support.

**Researchers should plan for sharing, thus reducing costs**

The use of shared protocols, measurement tools and metadata standards greatly reduces the burden of data standardisation. While researchers should not lose sight of local specificities, standardising core data collection tools at the outset will make data sharing cheaper, easier and more productive.

**Governance must be transparent, equitable and flexible**

Clear, concise terms of data submission and use can help erode the fear many researchers still have of sharing data. While more restrictive measures may be necessary to build trust, flexible terms should allow greater sharing as experience grows and norms change.

**Informatics systems, tools and governance structures can be shared**

WWARN has developed good systems for the automated ingest and management of heterogeneous malaria data; community meta-data standards and systems for developing them; and clear governance structures. All of these can be adapted for other diseases. It's important, however, to reconsider the purpose and users of the new platform. Governance structures designed for platforms supporting pooled analysis of post-publication data by academics will not serve the needs of platforms aiming to provide real-time surveillance data.

**Disease expertise remains necessary**

While much can be shared across platforms, deep knowledge of specific disease fields is necessary at the platform design stage, as well as to guide useful analysis.

WWARN founders envisaged a “comprehensive efficacy and resistance database [which] will provide malaria control managers, surveillance programs and policymakers with prompt access to up-to-date evidence of temporal and geographic trends in antimalarial drug resistance at the global scale ^24^”. We found that the institutional and professional incentives prevailing at the time made it impossible to achieve this goal. However, once the Network aligned its practices with prevailing incentives, it proved both the feasibility of compiling and standardising individual patient and parasite data across hundreds of studies, and the utility of pooled analyses in guiding treatment policy.

The hurdles WWARN initially faced were the product of archaic academic norms, coupled with a dysfunctional global health architecture. On the 'supply' side, academic researchers were unwilling to risk sacrificing publications (and thus promotions) by providing pre-publication data. Government researchers did not supply data in deference to WHO officials, who were unsupportive because they were concerned that the venture duplicated their own work. On the 'demand' side, the academic researchers who conceived of the platform made assumptions about policy-makers' needs, without actually consulting them widely. In a 'pivot' similar to those performed by many successful ventures in the information technology sector ^27, 28^, WWARN then reformulated its goals, aligning them to the prevailing incentives while side-stepping institutional obstacles. Rather than focusing on the active surveillance of antimalarial resistance, the Network turned its attention to generating new learning from pooled analyses – arguably a goal with more immediate value for millions of patients around the world. In the course of developing this more research-centred platform, WWARN has developed many systems, tools and procedures that can be extended to platforms aggregating individual patient data for other diseases. Indeed, that process has begun.

The WWARN experience underlines the extent to which current incentives in academia run counter to the open sharing of information. In theory, pooling data from large numbers of similar clinical trials provides an opportunity to unearth new knowledge more rapidly, and several consortia and closed research collaborations stand as proof-of-concept of the utility of sharing data collected in low-income settings ^16, 29, 30^. Like many other collaborations, WWARN was able to attract contributors only after it committed to a model that allowed contributors to restrict access to their data, and rewarded them with authorship on publications likely to be highly cited. By adopting the study group model, which appealed to data contributors, WWARN was able simultaneously to build up the database and to begin to conduct important pooled analyses that have contributed directly to improvements in global policy.

Broader consultation in the planning stages of WWARN may have revealed as somewhat unrealistic the initial goal of short-circuiting cumbersome publication processes to get actionable data into the scientific commons more rapidly. However, the network should be applauded for changing its strategy; WWARN was in no position by itself to change the personal and institutional incentives that stand as obstacles to the true potential of data sharing. If that potential is to be achieved, the publication of papers in peer reviewed journals must lose their pre-eminence as a measure of scientific productivity in academia. We believe that depositing data in well-curated, quality-assured databases should be rewarded professionally just as publication of papers in high impact journals now is. The use of data in a pooled analysis that demonstrably changes policy should be rewarded at least as well as a citation in a journal. Outside of academia, there is a need to reform the institutional relationships and funding mechanisms that create rivalry and open competition between organisations who at least nominally share the goal of increasing the flow of high quality scientific evidence, and reducing disease and death.

Once it bowed to the prevailing incentive structures, the WWARN network published several pooled analyses which established the value of the resource as a source of additional learning. This has encouraged drug developers and global health organisations to begin to request analyses using the database, and has prompted changes to access policies to facilitate use of the resource by all legitimate analysts.

Most of the proposed analyses seek to inform policies which will be made at the global level. Global policies do often trickle down into country-level policy because health ministries in lower-income countries follow global guidance quite closely. For two decades, investigators have pointed out that those setting the questions in health research in tropical countries are very rarely natives of those countries, and almost never the people who could use research results to effect change ^31– 33^. Several interviewees in our study were active as both researchers and policy advisors; they tended to use international studies to bolster local findings which politicians find more immediately compelling. Where the divide between researchers and policymakers is more pronounced, our findings support the view that agenda-setting in global health research remains lop-sided, even in the context of a project that set out to be an international collaboration. One potential way of increasing the local utility and uptake of data would be to encourage more active participation in question-setting by national programmes, working together with the endemic country researchers who best understand the local realities.

However this may be hard to achieve in reality. National policy makers were under-represented in our study, in part because their close relationship with WHO has in many cases stood between them and direct contact with WWARN. The policy makers we spoke to stressed that they were consumed with day-to-day programme delivery. This was confirmed by several endemic country researchers who serve on national advisory panels; they noted that national programme managers had little band-width left for formulating research questions that did not relate directly to operations. In addition, policy makers were wary of pooled analyses, feeling that they were likely to obscure local realities.

Endemic-country investigators said they lacked the skills and the resources to initiate study groups themselves; with the exception of South Africans, few researchers from endemic countries have yet initiated an interrogation of the shared dataset. This mirrors the experience reported by the Alpha Network, a large consortium of HIV researchers which invests more heavily than WWARN in local skills-building ^34^. Between 2005 and 2015, Alpha held a dozen multi-country analysis workshops in various sub-Saharan African countries, each focused on a specific research question. An examination of the workshop descriptions on the Alpha website suggests that many of the topics are chosen because they are of interest to international modelling groups or UNAIDS ^35^. This supports anecdotal reports from Alpha network members and from an interviewee in our study with experience in a different HIV data sharing consortium, who said that researchers from participating sites have been slow to initiate secondary pooled analysis of potential relevance to local policy-makers.

This raises the possibility that large, pooled, multi-country datasets are of limited immediate value to national disease control programmes; the information they yield is likely to affect local programmes only through the mediation of higher-order “brokers” in global health. These brokers currently include the WHO, other global health organisations and well-resourced research groups run mostly by universities and other institutions in rich countries. In theory, there is no reason that endemic-country researchers should not share or take over the complex pooled analyses that fulfil the needs of these information brokers. Indeed, their superior understanding of the contexts in which data were collected and policies must be implemented mean they are well-suited to the task. However our study suggests that in most endemic countries, well-trained researchers are principally rewarded for collecting and analysing primary data. They felt that they - and many of their peers in low-income countries - lacked full access to the collaborative networks and technical skills that large pooled analyses require. Initiatives such as the INDEPTH network, which builds capacity for data management, sharing and analysis across demographic surveillance sites in 19 countries in Africa, Asia and Oceania, demonstrate how investment in data sciences can pay off. Targeted calls and prizes could provide further motivation ^36, 37^, but supporters of research may need to earmark a percentage of all funding for capacity building if they wish to redress structural imbalances in biomedical knowledge generation and use.

We recommend exploring this dynamic empirically, by actively incentivising locally-led, policy-relevant secondary analysis using data from WWARN and other similar resources, then systematically tracking the use of any research results in local policy formulation.

Sharing data with a view to generating new, actionable information through pooled analyses requires investment in data curation, and in developing workable data governance structures. When WWARN took on this challenge they had no model to follow, and the investment was very substantial in terms of both time and money. This is often the case with pioneering ventures in science. Although the effort was of an entirely different order of magnitude, we note that it took 13 years and cost US$2.7 billion to arrive at the first draft sequence of the human genome. Structural changes to incentives and widespread sharing of methods and tools have brought those costs down to under US$1,500 since 2008 ^38^. On a far smaller scale (and with less outside help to change incentives that obstruct sharing), but somewhat analogous nonetheless, WWARN's procedures, informatics tools and policies are now being adapted for other diseases far more quickly and cheaply. The network has developed a solid foundation on which other data sharing infrastructure can be built; research funders and other investors in global health have an interest in supporting the group to share its experience more widely with other academic researchers and beyond.

Our study has several limitations. It was modest in scope, focusing on just one data sharing initiative. Clearly, the experience of a single, well-financed group embedded in a well-known academic institution, led by passionate scientists dealing with a subject considered to be of some urgency in global health, will not be relevant to all other settings and diseases. No past or present employee or close associate of WWARN was involved in data analysis or interpretation for this study, though some had an opportunity to review a late draft of the manuscript for accuracy. Employees of the network provided access to documentation for the records review, as well as suggesting an initial group of people for interview. It is possible that this introduced bias to our study. National malaria programme managers are clearly under-represented, for example, in part because of the firewall between WWARN and national programmes created by the 2009 memorandum of understanding with WHO. However, we did interview several people who chose not to be involved with WWARN as well as several former staff; the range of views expressed suggested that we achieved a well-rounded view of the network. To comply with the terms of participant consent, and because of time constraints, all interviews were coded by a single investigator (EP). This forecloses inter-coder validity checks. The interpretation of other information, including the records review and the witness seminar, was, however, discussed in detail between the authors.

The WWARN experience suggests that truly useful data sharing platforms must be thought of as long-term, infrastructural investments; they cannot be thrown up as rapid, project-based responses to funder or journal demands. To succeed, a data platform must consult widely at the outset with all potential platform users. It must align incentives and governance structures to meet the needs of data contributors, likely users, platform administrators and funders. If it is to maximise its potential impact on health, a platform must standardise data and actively facilitate the analysis of pooled data to answer locally relevant questions.

Though conceived to generate information about malaria resistance, the true value of WWARN may be in the lessons it has provided about the challenges of sharing data in an environment where pressure to share still outweighs experience. Key among these lessons is that data aggregators do not, by themselves, have much influence over the incentives that shape the data sharing landscape. It is up to the wider research community - including public and private funders, academia, industry and publishers - to reshape that landscape so that scientists, physicians and public servants are rewarded for sharing data and information towards a common goal.

## Data availability

The data referenced by this article are under copyright with the following copyright statement: Copyright: © 2017 Pisani E and Botchway S

Data related to this study can be found in the Harvard Dataverse repository, under a CC0 licence, available at doi: 10.7910/DVN/V1TKIO ^20^.

The files include:

- Original study protocol with question guide and consent forms
- Completed COREQ checklist for reporting qualitative data
- Coding tree for NVivo data coding of interviews and documents
- Bibliography of WWARN-related papers, in downloadable .ris format
- Spreadsheet of WWARN-related papers with first and last authors, institutional affiliations, number of authors, in .tab format for download to Excel and other programmes.

The document review included published papers, conference presentations and material published by funders or the WWARN platform on their websites. Citations are given for those materials that are in the public domain. Also included were documents that are not in the public domain, and which the authors of this paper are not legally authorised to make available. These include the internal records of WWARN's board and scientific advisory committee meetings, minutes from meetings with WHO and other partner institutions, and correspondence between Oxford University, the Bill and Melinda Gates Foundation and other institutions. While these were made available to researchers for analysis, permission was not granted to publish documents relating to individuals who did not, at the time of the interaction in question, grant consent to be identified. A list of the documents were coded in NVivo but can not be shared, along with their dates and content type (minutes of meetings, internal correspondence etc.) is available in the Dataverse repository cited in this statement. The terms of consent for interviews (also in the repository) also preclude the publication of interview tapes or transcripts. This step was taken to allow interviewees to speak openly about events which involve their current or former employers or employees, research funders or grantees, and their scientific colleagues and rivals, without fear of reprisal. The Oxford Tropical Research Ethics Committee accepted these provisions, and further required that a list of potential interviewees be removed from the study protocol, to further protect anonymity. The authors will be happy to run specific queries derived from the coding tree, and provide appropriately anonymised/redacted results from the records review and interviews to readers on request. Please send query requests stating your institutional affiliation and the reason for the request to Oxford Tropical Research Ethics Committee (oxtrec@admin.ox.ac.uk) quoting reference 593-16. The ethics committee will pass those requests it considered reasonable to the corresponding author for execution.

## Ethical statement

The study was approved by the Oxford Tropical Research Ethics Committee (OxTREC Reference: 593-16). For administrative reasons, research funding for both EP and SB was channelled through Oxford University and the Infectious Disease Data Observatory, an umbrella organisation that includes the WWARN platform. WWARN staff had the opportunity to comment on the research protocol, as did Oxford University's Tropical Research Ethics Committee, which provided ethical approval for the study. Neither WWARN nor the ethics committee suggested any major alterations to the study plans.
