# Supplementary material for: Sharing individual patient and parasite-level data through the WorldWide Antimalarial Resistance Network platform: A qualitative case study
Source: Wellcome Open Res. 2017 Aug 16;2:63. [Version 1] doi: 10.12688/wellcomeopenres.12259.1 (PMC5627501; doi:10.12688/wellcomeopenres.12259.1)
Supplement: Peer review history

# Peer review: Referee response for version 1

**DOI:** 10.21956/wellcomeopenres.13272.r25804

**Published:** 2017-10-03

**Authors:** Daniel Mietchen

**Affiliations:** 1 Data Science Institute, University of Virginia, Charlottesville, VA, USA

This article investigates the sociocultural context of the establishment, design and development of a global epidemiological data sharing initiative — the WorldWide Antimalarial Resistance Network (WWARN) — from its beginnings over a decade ago until about a year ago.

 It highlights that

- data sharing requires dedicated infrastructure, including relevant standards and standard-aware tools
- designing this infrastructure and the associated policies requires engagement with all relevant stakeholders
- developing the above takes time and requires stable funding, including for the stakeholders involved
- buy-in from stakeholders additionally requires significant cultural changes within the research and policy ecosystem in terms of how data sharing and the reuse of shared data and tools are valued (especially in epidemiology, but also beyond).

None of these points are particularly new, but spelling them out on the basis of a concrete example like WWARN and enriching them with sound bites — e.g. “Seven years ago, "data sharing" was a swear word.” — is useful both for conversations within a given field (in this case epidemiology) and across fields.

 The main strength of the paper lies in weaving in the often-neglected concept of equity, e.g. by giving voice to representatives from malaria-endemic countries (i.e. mostly in lower and middle income settings) and by discussing

- the need for capacity building in such countries in terms of analyzing and otherwise utilizing data pooled across multiple countries
- the various direct and indirect interactions between single-country and multi-country studies
- the role of mediators like the World Health Organization
- the role of WWARN study sections as well as
- some generic and specific inequalities in research funding, publishing and assessment.

The three main sources of the information presented are

- a set of documents from WWARN and its partners as well as individuals involved
- a set of interviews with individuals who are or have been involved with WWARN internally or externally
- a witness seminar that brought some of those individuals together to discuss themes emerging from preliminary analysis of the documents and the set of individual interviews.

Basically none of these key sources have been shared (for justified privacy reasons), which naturally limits the reproducibility of the study. However, the authors document their sources' metadata in sufficient detail to allow the reader to follow and evaluate the logic of the overall narrative as well as some of its highlighted details, e.g. the differences of perspectives of the different stakeholders, and the change of focus of the network from policy-oriented to research-oriented.

 The study was qualitative in nature, so there is basically no statistical analysis. It also looked at the WWARN case study almost in isolation, which limits the generalizability of the conclusions in principle, but as WWARN's approach itself is now being generalized to other epidemiological use cases in the framework of the newly established Infectious Diseases Data Observatory (IDDO, a WWARN umbrella), this provides fertile ground for testing the validity of the paper's conclusions in other epidemiological contexts like schistosomiasis, Ebola and visceral leishmaniasis.

 Overall, I found the paper well written, and in the few cases where I found something that would benefit from corrections, additions or clarification, I left a comment as part of my annotations of the paper, which are accessible via https://via.hypothes.is/https://wellcomeopenresearch.org/articles/2-63/v1 . None of these would preclude approval here in Wellcome Open Research, but I would still like to encourage the authors to address them in a revised version of the manuscript.

 One point I did not mention in my annotations is the lack of visuals. While not essential, it would have been useful to have some visual representation of the kinds of data that are now shared through WWARN/ IDDO, as well as of the way(s) in which they were shared (privately or more broadly) before WWARN, over the course of WWARN development and now through the WWARN/ IDDO system. This can help the reader, but it can also help to attract new readers from within or near the paper's target group of "researchers who share data, or are contemplating doing so".

 For my notes on drafting this review, see https://github.com/Daniel-Mietchen/ideas/issues/494 .

 This review is licensed CC0, as per http://creativecommons.org/publicdomain/zero/1.0/deed.en .

I have read this submission. I believe that I have an appropriate level of expertise to confirm that it is of an acceptable scientific standard.

# Peer review: Referee response for version 1

**DOI:** 10.21956/wellcomeopenres.13272.r25063

**Published:** 2017-09-06

**Authors:** Kobus Herbst

**Affiliations:** 1 Africa Health Research Institute, Durban, South Africa

This is a well researched and written report on the complexities involved in sharing public health data. The investigators went to considerable lengths in researching the process leading up to and including the actual sharing of WWARN data.

 Although the authors acknowledge the limitation of the study as being limited to only data sharing initiative, the study nevertheless provides several valuable insights into the complexities involved in data sharing initiatives of this kind.

 Their findings certainly echo my own experience with sharing INDEPTH data through the Wellcome-funded iSHARE2 inititiative.

I have read this submission. I believe that I have an appropriate level of expertise to confirm that it is of an acceptable scientific standard.
